# Coevolution of Cyanogenic Bamboos and Bamboo Lemurs on Madagascar

**DOI:** 10.1371/journal.pone.0158935

**Published:** 2016-08-17

**Authors:** Daniel J. Ballhorn, Fanny Patrika Rakotoarivelo, Stefanie Kautz

**Affiliations:** 1 Department of Biology, Portland State University, 1719 SW 10th Ave, Portland, OR 97201, United States of America; 2 UMR PVBMT, Université de La Réunion, 15 Avenue René Cassin, 97715 Saint-Denis, Réunion, France; Northern Illinois University, UNITED STATES

## Abstract

Feeding strategies of specialist herbivores often originate from the coevolutionary arms race of plant defenses and counter-adaptations of herbivores. The interaction between bamboo lemurs and cyanogenic bamboos on Madagascar represents a unique system to study diffuse coevolutionary processes between mammalian herbivores and plant defenses. Bamboo lemurs have different degrees of dietary specialization while bamboos show different levels of chemical defense. In this study, we found variation in cyanogenic potential (HCNp) and nutritive characteristics among five sympatric bamboo species in the Ranomafana area, southeastern Madagascar. The HCNp ranged from 209±72 μmol cyanide*g^-1^ dwt in *Cathariostachys madagascariensis* to no cyanide in *Bambusa madagascariensis*. Among three sympatric bamboo lemur species, the greater bamboo lemur (*Prolemur simus*) has the narrowest food range as it almost exclusively feeds on the highly cyanogenic *C*. *madagascariensis*. Our data suggest that high HCNp is the derived state in bamboos. The ancestral state of lemurs is most likely "generalist" while the ancestral state of bamboo lemurs was determined as equivocal. Nevertheless, as recent bamboo lemurs comprise several "facultative specialists" and only one "obligate specialist" adaptive radiation due to increased flexibility is likely. We propose that escaping a strict food plant specialization enabled facultative specialist bamboo lemurs to inhabit diverse geographical areas.

## Introduction

Antagonistic interactions between herbivores and plants, parasites and their hosts as well as predators and prey can be driven by escalating co-evolutionary arms races, in which the focus of selection on the host or prey is to escape the interaction, and the focus of selection on the enemy is to overcome those escape strategies or defenses [1–3]. In plant-herbivore systems, the result can be sophisticated arsenals of mechanical and chemical defenses in plants and counter-defense mechanisms ranging from behavioral to physiological adaptations in herbivores [4–6]. Although the evolution of physiological adaptations of herbivores (physiological specialists) to overcome specific toxic constituents in their host plants is commonly observed (i.e. increasing specialization), the opposite direction may also evolve through the development of a more generalist foraging strategy (behavioral generalists) [7,8]. By using a generalist foraging strategy, herbivores can reduce the negative impact of particular toxins in specific plant species by "diluting" these toxins as they utilize a broader range of different food plant species [9,10]. Another potential advantage of generalization over specialization is the expansion of the diversity of suitable host plants and potentially larger spatial distribution ranges [11].

Coevolutionary processes between plant defenses and herbivores have been described in detail for some groups of herbivores, particularly in insects [12–15]. Such processes have been little studied in other animal groups in comparison [16,17], including mammalian herbivores (but see [6,18–22]). In contrast to phytophagous insects, which often feed on only one plant family or genus, dietary specialization is considered the exception rather than the rule in vertebrate herbivores [9,23–30]. In fact, 98% of mammalian herbivores are generalist feeders [31–33], whereas across all herbivorous insects, it is estimated that <10% feed on plants in more than three different plant families [4,34]. Traditionally, ecologists have classified herbivores as specialists only if they consume one or a small number of different food items in their native habitat (i.e., a limited realized diet) [23,24]. Recently, many ecologists have defined a specialist herbivore as one that displays unique physiological (*Heliconius sara* [35]), behavioral, or morphological adaptations (*Zygaena filipendulae* [36]) to consume what Robinson and Wilson [37] refer to as an intrinsically "difficult" diet. A difficult diet is one that is not commonly used by other herbivores because of chemical or physical characteristics that make it generally unpalatable or of low nutritional value [27,38,39]. "Obligate specialists" always have a narrow food range of difficult food items and show morphological adaptations and/or the loss of redundant behavioral flexibility precluding them from expanding their diet under changed environmental conditions. "Facultative specialists" have a consistently narrow range of food sources during at least one spatial or temporal scale, but are able to expand their diet to include less difficult foods when environmental conditions allow. "Facultative generalists" are able to consume a wide variety of foods. However, they may occasionally demonstrate a narrow food range on less difficult plants in a similar manner to specialists. "Obligate generalists" always have a wide realized niche because of a relatively narrow fundamental niche, precluding them from eating much of any difficult plant [39].

Bamboo lemurs of the genus *Prolemur* and *Hapalemur* are herbivores, which primarily feed on a range of bamboo species [40,41]. However, species of the genus *Hapalemur* can also feed on alternative plants as primary food source in areas where bamboo is absent [42]. In the Ranomafana area in Southeast Madagascar, three species of bamboo lemurs (greater bamboo lemur, *P*. *simus*; golden bamboo lemur, *H*. *aureus*; and gray bamboo lemur *H*. *griseus*) occur sympatrically. These lemurs show different degrees of food plant specialization varying from obligate specialists to facultative specialists. At this site, five bamboo species (*Cathariostachys madagascariensis*, *Cathariostachys capitata*, *Nastus elongatus*, *Cephalostachyum* sp., and *Bambusa madagascariensis*), with different levels of cyanogenic chemical defense, serve as food plants for the bamboo lemurs. Existing interactions between the lemurs and bamboo species have repeatedly been observed under natural field conditions [40, 43–46]. In this system, the greater bamboo lemur represents the most specialized herbivore. This lemur species lives almost entirely on a single bamboo species, giant bamboo (*C*. *madagascariensis*), which accounts for more than 95% of its diet [40] and therefore can be considered an obligate specialist. The other two lemur species, the golden and the gray bamboo lemur also rely heavily on *C*. *madagascariensis*, which in our study area constitutes 78% and 72% of their diets, respectively [40]. However, throughout their distribution range these species make regular use of other bamboos as well as various plant species, even from different families, making them facultative specialists. In particular, *H*. *griseus* and its subspecies (which have recently been elevated to species status and are referred to as *H*. *occidentalis*, *H*. *meridionalis*, *H*. *alaotrensis*, and *H*. *gilberti*; [47]) occur in various habitats ranging from littoral forests to swamps that contain little or no woody bamboo. In their habitats, the lemurs feed on a range of different plant species [42] and thus obviously are able to expand their diet to include less difficult foods when environmental conditions allow. These lemurs do not occur in the Ranomafana area.

Giant bamboo (*C*. *madagascariensis*) contains > 200 μmol cyanide per gram dry weight making it one of the most cyanogenic plants worldwide [48] and therefore clearly represents a difficult diet [40]. Plant cyanogenesis is defined as the enzymatically accelerated release of highly toxic hydrogen cyanide from preformed cyanogenic compounds–mostly cyanogenic glycosides–in response to cell damage [49]. Toxicity of cyanide to vertebrates mainly is due to the inhibition of the mitochondrial respiration pathway by blocking the cytochrome a/a3 dependent oxidase. Furthermore, cyanide blocks the oxygen binding site in hemoglobin, thus, reducing oxygen transport capacities in blood [50]. Beyond these major toxic actions of cyanide, the activity of many other metal-containing enzymes (e.g. peroxidases, catalases) is inhibited as the cyanide ion binds to their active center [50]. In particular, the inhibition of cellular respiration caused by cyanide is a general mechanism making this chemical toxic to all eukaryotes. Consequently, cyanogenesis is considered an effective plant defense against multiple groups of herbivores [51–54]. As the young shoots of *C*. *madagascariensis*, which are a favored food source of the lemurs when seasonally available, show exceptionally high cyanide concentrations [48], the cyanide uptake by *P*. *simus* is arguably the most extreme case of regular cyanide intake ever described for mammals. Based on plant cyanide content and the amount of plant material consumed, the lemurs ingest up to 48 times the lethal cyanide dose of an average mammal per day [48].

Due to the availability or quality of host plants, herbivorous food specialists often show restrictions in their distribution range [55,56]. In this line, the present-day distributions of both the greater bamboo lemur and the golden bamboo lemur are strongly restricted. The golden bamboo lemur occurs in the rainforests of southeastern Madagascar including the Ranomafana National Park and further south in the Andringitra National Park as well as in the corridor between these areas [43,57–58] and possibly northeast to the region of Betsakafandrika [59]. The distribution of the greater bamboo lemur, which shows the highest food specialization, is even more restricted. The distribution range includes the south-central portion of the country’s eastern rainforests at elevations of 200–1,100 m [60,61]. Like the golden bamboo lemur, this species occurs in the Andringitra National Park and in our study area, the Ranomafana National Park. Furthermore, the species was found in rainforests in the region of Andasibe/Perinet and in the forest of Maromizaha [41,62–64]. However, in contrast to their current limited distribution, historical records and subfossils confirm a formerly widespread occurrence of *P*. *simus* that covered wide areas of Madagascar [65]. Compared to the highly specialized greater and golden bamboo lemurs, the less specialized gray bamboo lemur (*H*. *griseus*) faces a lower risk of extinction. *Hapalemur griseus* shows a wide distribution range throughout the remaining forests of eastern Madagascar from the Tsaratanana Massif and an area south of Maroantsetra in the far north to Fort Dauphin in the far south [66].

The evidence that―in addition to human impact―food specialization limits distribution range and potentially incurs a higher risk of isolation and ultimately extinction leads to the question of whether food specialization is an ancestral or derived trait in bamboo lemurs. To better understand the potential role of food quality for the evolution of specialization, we asked four questions: (1) Are there quantitative differences in cyanogenic potential (HCNp; concentration of cyanogenic precursors) among different bamboo species? (2) Is there covariation of cyanide and soluble protein in bamboo shoots as a representative nutritive trait (3) How did HCNp in bamboos evolve? (4) Did bamboo lemurs evolve towards a higher specialization or towards expansion of food plant use? To address these questions, we compared quantitative data on HCNp and soluble protein concentration of *C*. *madagascariensis* as well as four other bamboo species (*C*. *capitata*, *N*. *elongatus*, *Cephalostachyum* sp., *B*. *madagascariensis*), which serve as food plants for the three bamboo lemurs in southeastern Madagascar in and around the Ranomafana National Park. We then mapped the HCNp of these bamboo species on a phylogeny to test our hypothesis of an evolutionary increase in cyanide concentration in bamboos. Finally, we tested whether lemur evolution is driven by increased specialization or relaxation of host plant specificity.

## Materials and Methods

### Ethics Statement

The bamboo species collected for this study are not threatened species. Plant samples were collected outside protected areas of Ranomafana National Park (permit N°020/08/meeft/sg/dgef/dsap/sse as obtained by the ministere de l’environment, des eaux et forets et du tourisme). The conducted research is in compliance with laws and ethical standards of the countries in which research was conducted.

### Study Site and Plant Material

Field studies on Madagascar were conducted in January-February 2008. Study sites were located in the southeast of Madagascar with the main study site being in the vicinity of the Ranomafana National Park, Fianarantsoa (~21°15´ S and 047°25´ W, elevation 1000 m). The four bamboo species *C*. *madagascariensis*, *Cephalostachyum* sp., *N*. *elongatus*, and *B*. *madagascariensis* were collected at this site. Samples of *C*. *capitata* were collected in nearby Kianjavato (~21°21´ S and 047°45´, elevation 300 m). See S1 Table for detailed information on sample location, sampling date, and voucher specimen deposition.

**Fig 1:**
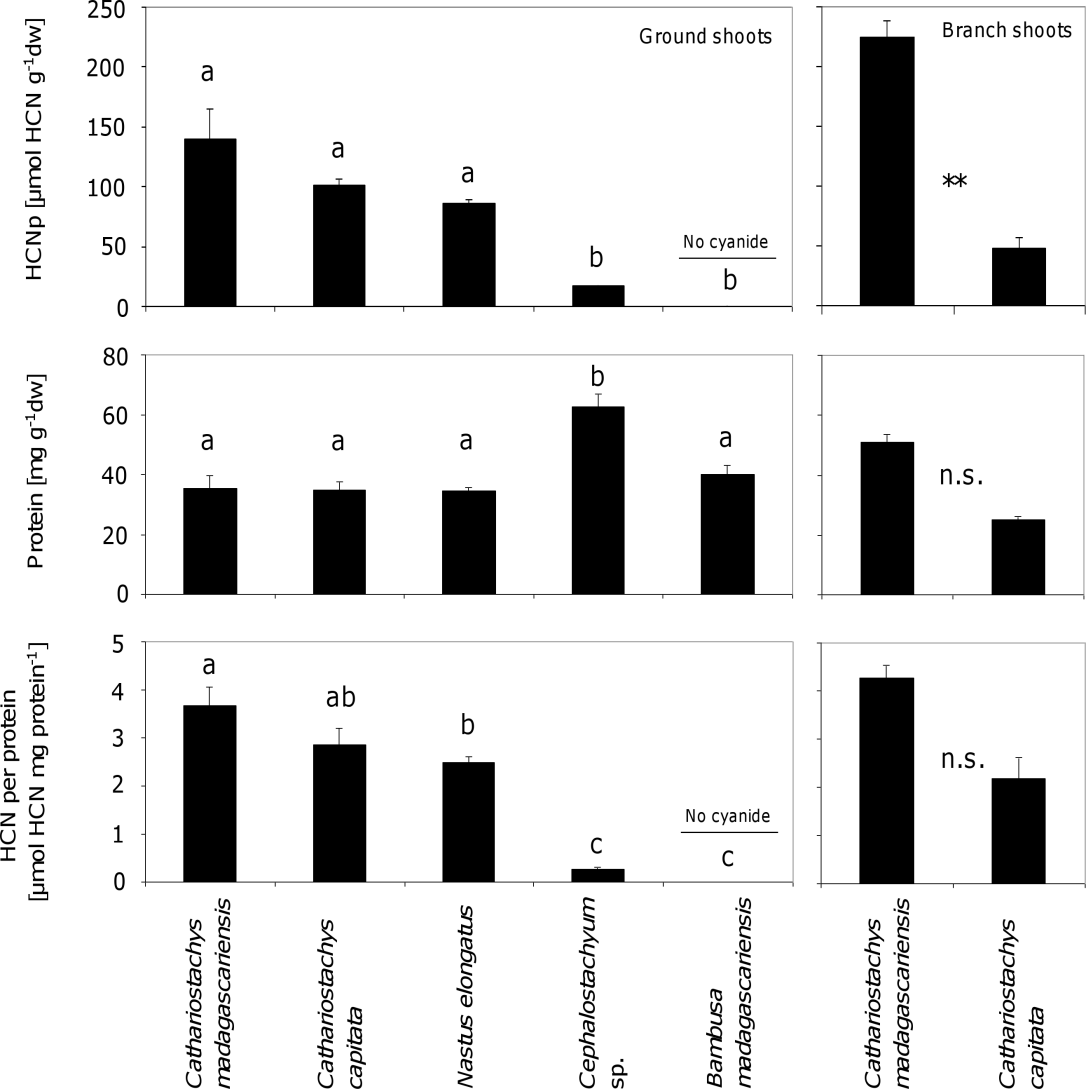
Cyanogenic potential, protein concentration and cyanide per protein ratio in shoots of sympatric bamboo species in the Ranomafana area. Ground shoots of five (left column) and branch shoots of two (right column) different bamboo species serving as food plants for bamboo lemurs in southeastern Madagascar were analyzed for their cyanogenic potential (HCNp; amount of cyanogenic precursors), concentration of soluble protein, and the nutritionally important ratio of cyanide per protein. Bars are means ± SE. Different small-typed letters above the bars represent significant differences according to post-hoc analysis (Tukey’s HSD, P < 0.05) after one-way ANOVA. Asterisks (**P < 0.01) in the right column represent significant differences between traits according to t-tests, whereas “n.s.” means no significant differences.

Young shoots of all five bamboo species collected in the field were analyzed for cyanogenic potential (HCNp = concentration of cyanogenic precursors) and concentration of soluble proteins (see below). In *C*. *madagascariensis* and *C*. *capitata*, we differentiated between two types of shoots: i) ground shoots, i.e. shoots growing from subterraneous rhizomes and ii) branch shoots located up to 15 m above ground in the canopy area of mature leaf-carrying shoots (Fig 1), whereas the other bamboo species showed no branch shoots at time of analysis. Shoots selected for analysis had freshly developed during the rainy season (December-March). At time of collection, ground shoots were 30 cm to 50 cm in height, whereas branch shoots were 10 cm to 30 cm in length. Samples were collected outside protected areas of Ranomafana National Park but within lemur forage areas according to observed feeding damage on bamboo shoots and branches or leaf remains beneath bamboo plants.

### Bamboo Chemistry

#### Sampling

In the present study, we focused on young shoots of bamboos as these plant parts represent a central component of the diet of bamboo lemurs. We quantified the cyanogenic potential (HCNp) as the most characteristic toxic trait in bamboo. In addition, we quantified soluble protein concentration as an important nutritive trait [67]. To avoid a premature release of HCN due to damage or degradation processes of plant tissues, shoots were sampled quickly in the morning. Injury of shoots during collection and transport was avoided and entire shoots were transported to the field lab. In the field laboratory, we measured quantitative variation of both traits in ground shoots and branch shoots (if available), and we considered ontogenetic variability of traits by sampling defined parts of the shoots, which in our previous study proved to be a good estimate for the overall cyanide concentration in the shoots [48]. Shoots were cut lengthwise and shoot material was collected with a cork borer (9 mm in diameter). Each sample was then cut lengthwise with a razor blade exactly in the middle. One part was used for nutritional analysis in the field and the other was dried at 75° C in a drying oven until constancy of weight and weighed to the nearest 0.001 g. Both HCNp and protein are referred to in dry weight. Branch shoots were treated equally to ground shoots.

### Quantification of HCNp

The cyanogenic potential (HCNp) of shoot samples was measured by extraction and subsequent enzymatic degradation of cyanogenic precursors from plant material according to Ballhorn et al. [48]. Directly before analysis, plant material was ground for extraction of cyanogenic precursors with a mortar and pestle, which were kept on ice. Ice-cold 0.067 mol l^-1^ disodium hydrogen phosphate (2 ml g^-1^ fwt) and small amounts of sterilized sea sand (Sigma Aldrich, Deisenhofen, Germany) were added. The homogenized samples were filtered using 5 ml PE syringes (B. Braun AG, Melsungen, Germany) supplemented with cotton and the filtrate was used immediately for further HCNp and protein analysis.

For enzymatic degradation of the cyanogenic precursors, exogenous *β*-glucosidase from almonds (FlukaChemie AG, Buchs, Switzerland) in phosphate-citrate buffer (McIlvaine buffer), pH 5.6, was added to the respective sample in an amount that corresponded to 20 nkat. An enzyme activity of 1 kat (katal) is defined as a substrate conversion rate of 1 mol substrate per second under standard temperature and pressure [68]. Activity of *β*-glucosidase was determined by using *p*-nitrophenyl-*β*-D-glucopyranoside (Merck KGaA, Darmstadt, Germany) as an artificial chromogenic substrate. Thunberg vessels were used as reaction flasks for the determination of HCNp [69]. These vessels were sealed by a glass stopper with a side bulb (volume of about 5 ml). Thus, the vessels contained a closed headspace and the released HCN could not leak from the preparation. The mixture for incubation consisted of 0.05 ml supernatant of the filtered sample, 0.45 ml 0.067 mol l^-1^ aqueous sodium dihydrogen phosphate solution, 0.10 ml *β*-glucosidase solution, and 0.60 ml 0.2 mol l^-1^ NaOH in the side bulb of the stopper. This mixture was incubated in a water bath for 25 min at a temperature of 30°C. The enzymatic reaction was stopped by the addition of the NaOH solution, which was added from the side bulb of the stopper to the incubation mixture. By adding NaOH, the sodium salt of HCN was formed, which then was spectrophotometrically quantified using the Spectroquant^®^ cyanide test (Merck).

The standard preparation for spectrophotometric measurement of cyanide consisted of one aliquot (0.025 ml of shoot extract) that was taken from the stoppered incubation mixture. The sample was neutralized by adding 0.1 mol l^-1^ HCl (0.025 ml) and made up to a total volume of 5 ml by adding ddH_2_O. The concentration of the chromogenic product (polymethine; in the sample one mol of formed polymethine dye (Spectroquant® cyanide test) corresponds to 1 mol cyanide) was measured spectrophotometrically after 5 min of incubation time at a wavelength of 585 nm (Genesys 20, Thermo Spectronic, Madison, WI, USA). Quantification of cyanide in leaf samples was conducted using a calibration curve prepared from KCN solutions (in 0.067 mol l^-1^ aqueous sodium dihydrogen phosphate buffer) ranging from 0 to 1 mmol CN^-^ per liter.

### Quantification of Soluble Proteins

Samples were analyzed for concentration of soluble proteins according to Bradford [70]. The Bradford reagent (Biorad Laboratories, Munich, Germany) was diluted 1:5 with water and 20 μl of each sample were added to 1 ml of diluted Bradford solution. Bovine serum albumin (BSA; Fluka Chemie AG, Buchs, Switzerland) at different concentrations was used as standard [71]. After 5 min of incubation, the concentration of protein was spectrophotometrically measured at 595 nm (Genesys 20, Thermo Spectronic, Madison, WI, USA). We used the same individual plant extracts for protein measurements that were used for HCNp analyses. Thus, both traits could be quantitatively related to the same individual sample. The Bradford assay is suitable for estimating nutritive value in plants which are particularly rich in nitrogen-containing defense compounds as this assay is not measuring all nitrogen (as for example Kjeldahl which does not distinguish between the source of detected nitrogen), but only soluble and easily digestible proteins [67].

Quantitative effects of potential interference of plant phenolic compounds with plant protein during analyses were investigated in preliminary experiments in which individual plant samples were cut lengthwise while one subsample was analyzed with and the other without addition of polyvinylpolypyrrolidone (PVPP; Sigma-Aldrich, Buchs, Switzerland) before extraction [72]. PVPP serves as an effective absorbent for phenolic compounds [73]. Protein concentration under addition of PVPP was never higher than in samples not treated with PVPP indicating a limited impact of phenolics in bamboo on the digestibility of plant proteins (data not shown).

### Bamboo Phylogeny

#### DNA extraction and amplification

Total genomic DNA of all bamboo species was extracted from silica-gel-dried leaf material using the DNeasy® Plant Kit (Qiagen, Valencia, CA, USA) following the manufacturer’s protocol. The final elution of DNA was performed with 200 μl sterile water instead of AE buffer. A fragment of the chloroplast *rpl16* intron was amplified. Primers used for amplification were F71 (5’-GCTATGCTTAGTGTGTGACTCGTTG-3’) and R1661 (5’-CGTACCCATATTTTTCCACCACGAC-3’; [74]).

Polymerase chain reaction (PCR) was carried out in 25 μl reaction volumes consisting of 2.5 μl 10x PCR buffer (Roche), 2.5 μl dNTPs (at 2mM for each dNTP), 2.5 μl 10x Bovine Serum Albumin (BSA), 0.2 μl *Taq* Polymerase (Roche), 1.0 μl of each primer (10 μM), 11.3 μl ddH_2_O, and 4 μl of undiluted DNA-isolate. Thermal cycling parameters were: initial denaturation for 5 min at 95°C; 34 cycles of 95°C for 1 min, 55°C for 1 min, 72°C for 2 min; and a final elongation for 10 min at 72°C. After amplification, samples were kept at 4°C. Amplification products were viewed on 1% agarose gels (low melt) stained with ethidium bromide, and subsequently excised and purified using GELase enzyme (Epicentre, Madison, WI).

Fragments were sequenced using the Big Dye Terminator reaction kit (ABI PRISM, Applied Biosystems, Forster City, USA). For sequencing, the same set of primers was used as for PCR amplification in addition to the primers SAK8 (5’-CCATCCCACCCAATGAAG-3’) (http://www.eeob.iastate.edu/research/bamboo/pdf/PCR_protocols.pdf) and R1516 (5’-CCCTTCATTCTTCCTCTATGTTG’-3) [75]. Cycle sequencing was conducted with the following program: initial denaturation for 1 min at 96°C followed by 32 cycles of 96°C for 15 s, 50°C for 10 s, 60°C for 4 min. Sequence products were precipitated with 10 μl sterile dH_2_O, 2 μl of 3 m NaOAc, and 50 μl of 95% ethanol before they were loaded on an ABI 3730 (Applied Biosystems) automatic sequencer.

### Sequence Alignment

ABI traces were assembled with Geneious 5.4.3 [76] and manually adjusted. All sequences were unambiguous. The identity of sequences was verified using blast search [77]. Sequences were aligned using MUSCLE [78] as implemented in Geneious 5.4.3. Alignment parameters were default. In addition to sequences of 10 specimens (five species) generated in our lab, we downloaded a set of 46 sequences (representing 42 species from seven subtribes within the tribe Bambusoideae plus two outgroup taxa) from GenBank. We chose this single gene because of the taxonomic coverage in GenBank. No other genes were published with a similarly high representation at the time of analyses. Accession numbers and vouchers of all samples are given in Table 1 and S1 Table.

**Table 1:** Bamboo species and specimens included in the phylogenetic study, with GenBank accession numbers. All ingroup taxa belong to the tribe Bambuseae (woody bamboos) and subtribes are given. Sequences generated in this study are indicated in bold.

Species/Specimen	Subtribe	rpl16 intron GenBank accession no
*Arthrostylidium ecuadorense*	Arthrostylidiinae	AY912189
*Arundinaria gigantea*	Arundinariinae	U54742
*Arundinaria gigantea*	Arundinariinae	AF133465
*Atractantha radiata*	Arthrostylidiinae	AY912190
*Aulonemia fulgor*	Guaduinae	EF589613
*Bambusa longispiculata*	Bambusinae	AF133470
*Bambusa longispiculata*	Bambusinae	U54745
***Bambusa madagascariensis* H2**	**Bambusinae**	**KX528698**
***Bambusa madagascariensis* H3**	**Bambusinae**	**KX528696**
*Bambusa vulgaris*	Bambusinae	AY912192
*Buergersiochloa bambusoides*	outgroup	AF133461
*Cathariostachys madagascariensis* GB	Hickelinae	AY912202
***Cathariostachys madagascariensis* A1**	**Hickelinae**	**KX528694**
***Cathariostachys madagascariensis* B1**	**Hickelinae**	**KX528695**
***Cathariostachys capitata C2***	Hickelinae	**KX528690**
***Cathariostachys capitata C3***	Hickelinae	**KX528691**
***Cephalostachyum sp*. *F2***	Melocanninae	**KX528692**
***Cephalostachyum sp*. *G2***	Melocanninae	**KX528693**
*Cephalostachyum pergracile*	Melocanninae	AY912199
*Decaryochloa diadelpha*	Hickelinae	AY912203
*Eremocaulon asymmetricum*	Guaduinae	EF589615
*Eremocaulon aureofimbriatum*	Guaduinae	EF589616
*Glaziophyton mirabile*	Hickelinae	AF133471
*Glaziophyton mirabile*	Arthrostylidiinae	U54748
*Greslania circinata*	Hickelinae	AY912204
*Greslania rivularis*	Hickelinae	AY912205
*Guadua aculeata*	Guaduinae	EF589617
*Guadua amplexifolia*	Guaduinae	EF589618
*Guadua longifolia*	Guaduinae	EF589619
*Guadua paniculata*	Guaduinae	EF589620
*Guadua velutina*	Guaduinae	EF589621
*Hickelia madagascariensis*	Hickelinae	AY912206
*Nastus borbonicus*	Hickelinae	AY912207
*Nastus elatus*	Hickelinae	AF133469
*Nastus elatus*	Hickelinae	U54746
*Nastus elegantissimus*	Hickelinae	AY912208
*Nastus elongatus*	Hickelinae	AY912209
***Nastus elongatus* E1**	**Hickelinae**	**KX528697**
***Nastus elongatus* E2**	**Hickelinae**	**KX528698**
*Nastus productus*	Hickelinae	AY912210
*Olmeca recta*	Guaduinae	EF589622
*Olmeca reflexa*	Guaduinae	EF589623
*Oryza sativa*	outgroup	DQ289148
*Otatea acuminata*	Guaduinae	U54789
*Otatea acuminata*	Guaduinae	AF133474
*Oxytenanthera abyssinica*	Bambusinae	AY912193
*Perrierbambus madagascariensis*	Hickelinae	AY912211
*Phyllostachys pubescens*	Shibataeinae	AF133467
*Phyllostachys pubescens*	Shibataeinae	U54744
*Pseudosasa japonica*	Arundinariinae	AF133466
*Puelia olyriformis*	outgroup	AF133487
*Schizostachyum brachycladum*	Melocanninae	AY912200
*Schizostachyum luzonicum*	Melocanninae	AF133468
*Schizostachyum luzonicum*	Melocanninae	U54747
*Sirochloa parvifolia*	Hickelinae	AY912212
*Temburongia simplex*	incertae sedis	AY912214
*Valiha diffusa*	Hickelinae	AY912213

### Phylogenetic Analyses

For phylogenetic analyses, we used a Bayesian approach and a Maximum likelihood (ML) analysis as described previously [79]. Here, we adopted a conservative perspective and considered only those clades as well-supported that had a posterior probability of at least 0.95 and bootstrap support equal to or above 70%. The Bayesian (B/MCMC) analyses were performed using MrBayes 3.1.2 [80]. Posterior probabilities were approximated by sampling the trees using a Markov chain Monte Carlo (MCMC) method. The sequences were tested for the most appropriate model of DNA substitution analyses by the program MrModeltest version 2.3 [81]. Using AIC, GTR+ Γ was determined as the most appropriate maximum likelihood model of evolution for our dataset. MrBayes estimated the proportion of invariant sites, the gamma distribution shape parameter, base frequencies, and the substitution rates. No molecular clock was assumed. A run with 10,000,000 generations starting with a random tree and employing 4 simultaneous chains was executed. Every 100^th^ tree was saved into a file. The first 2,500,000 generations (i.e., the first 25,000 trees) were deleted as the “burn-in” of the chain. We plotted the log-likelihood scores of sample points against generation time using TRACER v1.5 (http://tree.bio.ed.ac.uk/software/tracer/) to ensure that stationarity was reached after the burn-in by checking whether the log-likelihood values of the sample points reached a stable equilibrium value [80]. Of the remaining 150,000 trees (75,000 from each of the parallel runs) a majority rule consensus tree with average branch length was calculated using the “sumt” option of MrBayes. Posterior probabilities were obtained for each clade.

The maximum likelihood (ML) analysis was performed with GARLI Version 0.951 [82] using default settings. Bootstrap support was based on 1,000 replications.

### Lemur Phylogeny and Ancestral States Reconstruction

Published nucleotide sequences for several mitochondrial genes (12s rRNA, cytochrome c oxidase subunit II (COII), cytochrome b (cyt-b), D-loop, as well as the Pastorini fragment (PAST) covering NADH3, NADH4L, NDH4 and 5 tRNAs) were obtained from GenBank (see Table 2 for accession numbers). These loci were chosen on the basis of taxonomic coverage; other candidate loci were discarded because of poor representation. In instances where a subspecies had recently been elevated to species level (within *Hapalemur*) the most recent names were adopted and the taxonomy, presented by Mittermeier et al. [47], was followed. We included a total of five (out of six recognized) *Hapalemur* species (*H*. *alaotrensis*, *H*. *aureus*, *H*. *griseus*, *H*. *meridionalis*, *H*. *occidentalis*), *Prolemur simus*, *Lemur catta*, three *Eulemur* species, two *Varecia* species from the Lemuridae family as well as three outgroup taxa from the Indriidae family (*Indri indri*, *Avahi occidentalis*, *Propithecus coquereli*). The choice of outgroup taxa was made in reference to previous studies of primate phylogeny [83].

**Table 2:** Lemur species and gene fragments included in the phylogenetic study, with family and GenBank accession numbers. Gene fragments: PAST, Pastorini fragment covering NADH3, NADH4L, NDH4 and 5 tRNAs; 12s rRNA; cyt-b, cytochrome; COII, cytochrome c oxidase subunit II; D-loop.

		Gene fragment				
Species	Family	PAST	12S	D-loop	cyt b	COII
*Avahi occidentalis*	Indriidae	AY582560	AF474241	AY584497	EF103291	AY584483
*Eulemur fulvus*	Lemuridae	NC_012766	NC_012766	NC_012766	NC_012766	NC_012766
*Eulemur macaco*	Lemuridae	NC_012771	NC_012771	NC_012771	NC_012771	NC_012771
*Eulemur mongoz*	Lemuridae	NC_010300	NC_010300	NC_010300	NC_010300	NC_010300
*Hapalemur alaotrensis*	Lemuridae	AF224576	AJ430037		AJ428969	
*Hapalemur aureus*	Lemuridae	AY582549	AF474239	AY584489	AY441446	AY515557
*Hapalemur gilberti*	Lemuridae					
*Hapalemur griseus*	Lemuridae	AY582551	AY582716	AY584490	HGU53574	AY569204
*Hapalemur meridionalis*	Lemuridae		AJ429205		AY441447	
*Hapalemur occidentalis*	Lemuridae	AY582553	AY582719	AY584492	AJ428982	AY569205
*Indri indri*	Indriidae	DQ856049	AY043340	DQ855966	AY441455	
*Lemur catta*	Lemuridae	NC_004025	NC_004025	NC_004025	NC_004025	NC_004025
*Prolemur simus*	Lemuridae	AY582548	AF474238	AY584488	AJ428977	AY569210
*Propithecus coquereli*	Indriidae	NC_011053	NC_011053	NC_011053	NC_011053	NC_011053
*Varecia rubra*	Lemuridae	AF224590	AF175791	AF173505	AY441450	VAEMTCOII
*Varecia variegata*	Lemuridae	NC_012773	NC_012773	NC_012773	NC_012773	NC_012773

Sequences of each mitochondrial protein-coding gene were aligned using amino acid sequences. For sequences of the D-loop region, ambiguously aligned regions were removed using Gblocks version 0.91b [84]. Models for DNA substitution were estimated for each gene in MrModeltest version 2.3 [81]. Using AIC, GTR+I+ Γ was determined as the most appropriate maximum likelihood model of evolution for the 12S, COII, cyt b, and PAST while the HKY+I+ Γ was determined the best fit maximum likelihood model for the D-loop region. The data set was partitioned into nine parts (12S, 1^st^, 2^nd^, 3^rd^ codon positions of COII, 1^st^, 2^nd^, 3^rd^ codon positions of cyt-b, D-loop, PAST). For each of the nine partitions, MrBayes estimated the proportion of invariant sites, the gamma distribution shape parameter, base frequencies, and the substitution rates (GTR model) or transition/transversion ratio (HKY model). Each partition was allowed to have its own model parameters as proposed by Nylander et al. [85]. Bayesian analyses, ML analyses using GARLI were performed exactly as described above for bamboo.

For the lemur phylogeny, we also conducted a maximum parsimony analysis (MP) with PAUP* [86] using the random stepwise addition option of the heuristic search for 500 replicates with tree bisection-reconnection (TBR) branch swapping, collapse of zero length branches, and equal weighting of all characters. A strict consensus was performed to summarize the results. To measure the robustness of branching patterns of the parsimony trees, bootstrap analyses (bs) [87,88] were executed by using the closest stepwise addition of the heuristic search for 500 replicates. Phylogenetic trees were drawn using TREEVIEW [89].

The lemurs’ degree of specialization was reconstructed based on the Bayesian inference of the lemur phylogeny. Three character states representing different degrees of specialization [0 = (obligate/facultative) generalists, 1 = facultative specialist, and 2 = obligate specialist] [27,38–39] were considered potential ancestral states. Ancestral states were reconstructed with maximum likelihood as the optimality criterion [90] on 1000 trees sampled with B/MCMC using the Trace Character Over Trees option in Mesquite 0.995 [91]. Using a likelihood ratio test, the asymmetric two-parameter model was selected for this analysis. Only ancestral states reconstructed with raw likelihood scores greater than 2.0 (i.e., the default setting T = 2.0 in Mesquite), corresponding to a conservative approximation of proportional likelihood values >0.95 in our analysis, were considered to be significant following Edwards [92].

### Statistical Analyses

Differences of ground shoot HCNp, protein concentration, and cyanide per protein among the five bamboo species included in this study were statistically analyzed using post-hoc analyses (Tukey’s HSD; P < 0.05) after one-way ANOVA. We tested for significant differences of the above-mentioned traits between branch shoots of *C*. *madagascariensis* and *C*. *capitata* using t-tests. Only these two species had developed branch shoots at the time of our fieldwork. All statistical analyses were carried out using the Statistical Package for Social Sciences (SPSS) 16.0 (SPSS for Windows, SPSS, Chicago, IL, USA).

## Results

### Cyanogenic Potential of Bamboos

Ground shoots of the different bamboo species showed significant differences in cyanogenic potential (HCNp) (F_4,20_ = 18.72; P < 0.001; Fig 1). *Cathariostachys madagascariensis* was the highest cyanogenic plant with concentrations of cyanide ranging from 68.7 to 223.8 μmol HCN*g^-1^ dwt in ground shoots. Shoots of *C*. *capitata* showed lower cyanide concentrations ranging from 64.7 to 150.6 μmol HCN*g^-1^ dwt in ground shoots, whereas ground shoots of *N*. *elongatus* and *Cephalostachyum* sp. contained lower amounts of cyanide ranging from 75.0 to 102.2 and 12.3 to 21.4 μmol HCN*g^-1^ dwt in ground shoots, respectively (Fig 1). HCNp among ground shoots of *C*. *madagascariensis*, *C*. *capitata* and *N*. *elongatus* showed no significant differences, whereas HCNp in *Cephalostachyum* sp. and *B*. *madagascariensis* was significantly lower (*B*. *madagascariensis* contained no detectable amounts of cyanide at all).

Cyanogenic potential in branch shoots of *C*. *madagascariensis* and *C*. *capitata* ranged between 110.2 to 328.5 and 19.4 to 98.6 μmol HCN*g^-1^ dwt, respectively. Branch shoots of *C*. *madagascariensis* had significantly higher cyanide concentrations compared to branch shoots of *C*. *capitata* (F = 12.33, T = 8.01, df = 30, P < 0.01).

### Soluble Protein in Bamboos

Concentration of soluble proteins showed significant variation among the bamboo species tested (according to one-way ANOVA; F_4,20_ = 12.20; P < 0.001). Protein concentration was significantly higher in ground shoots of *Cephalostachyum* sp. compared to ground shoots of the other bamboo species, which showed no significant differences among each other (Fig 1). In *Cephalostachyum* sp., protein concentrations ranged from 50.5 to 72.3 mg*g^-1^ dwt. *Bambusa madagascariensis* showed protein concentrations ranging between 30.3 and 50.9 mg*g^-1^ dwt. Protein concentrations in ground shoots of *C*. *madagascariensis* (23.3 to 46.4 mg*g^-1^ dwt), *C*. *capitata* (24.8 to 43.3 mg*g^-1^ dwt), and *N*. *elongatus* (32.6 to 38.5 mg*g^-1^ dwt) were similar to each other and were the lowest among the bamboo species analyzed. The amount of proteins in branch shoots of *C*. *madagascariensis* was higher when compared to the ground shoots and ranged from 33.4 to 76.0 mg*g^-1^ dwt, whereas protein concentration in branch shoots of *C*. *capitata* was lower when compared to ground shoots and showed values between 14.8 and 40.2 mg*g^-1^ dwt. Differences in protein concentration in branch shoots of *C*. *madagascariensis* and *C*. *capitata* were not significant (F = 0.56, T = 6.38, df = 30, P = 0.46).

### Cyanide per Protein

The nutritionally important ratio of HCN per protein in ground shoots showed significant variation [according to one-way ANOVA (F_4,20_ = 39.86; P < 0.001; Fig 1] and largely resembled patterns of cyanogenic potential (HCNp) among species. In ground shoots, the HCN per protein ratio showed highest values in *C*. *madagascariensis* ranging between 2.7 and 4.9 μmol HCN*mg^-1^ protein. Values in *C*. *capitata* were not significantly lower than in *C*. *madagascariensis* and ranged between 1.8 and 4.2 μmol HCN*mg^-1^ protein in ground shoots. Cyanide per protein ratios in ground shoots of *N*. *elongatus* ranged between 2.2 and 2.8 μmol HCN*mg^-1^ protein and were significantly lower than values for *C*. *madagascariensis* but did not differ significantly from *C*. *capitata*. *Cephalostachyum* sp. showed significantly lower HCN:protein ratios than *N*. *elongatus* ranging between 0.2 and 0.3 μmol HCN per mg^-1^ protein, whereas *B*. *madagascariensis* contained no cyanide (see above).

The cyanide per protein ratio in branch shoots of *C*. *madagascariensis* and *C*. *capitata* ranged between 2.5 to 7.3 and 0.3 to 5.0 μmol HCN*mg^-1^ protein, respectively. Differences in the HCN per protein ratio between branch shoots of *C*. *madagascariensis* and *C*. *capitata* were not significant (F = 0.09, T = 4.18, df = 30, P = 0.77).

### Host Plant Phylogeny

To generate a molecular phylogeny of bamboo, a total of 56 (10 new) sequences were used. A matrix with 1172 unambiguously aligned nucleotide position characters was produced for analysis. The alignment is available in TreeBASE (http://purl.org/phylo/treebase/phylows/study/TB2:S18996). The mean log likelihood of the Bayesian tree sampling was –3878.408 using the GTR + Γ model in MrBayes v3.1.1 [76] with 10,000,000 generations. The maximum likelihood search in GARLI v0.94 [78] resulted in a maximum likelihood tree with a final score of lnL = -3297.1615. Detailed information on base composition and estimated parameter values is given in S2 Table. The base composition of the chloroplast *rpl16* intron in the study species was highly AT biased (0.685), as is typical of chloroplast introns, and similar values were reported by Clark et al. [93].

**Fig 2:**
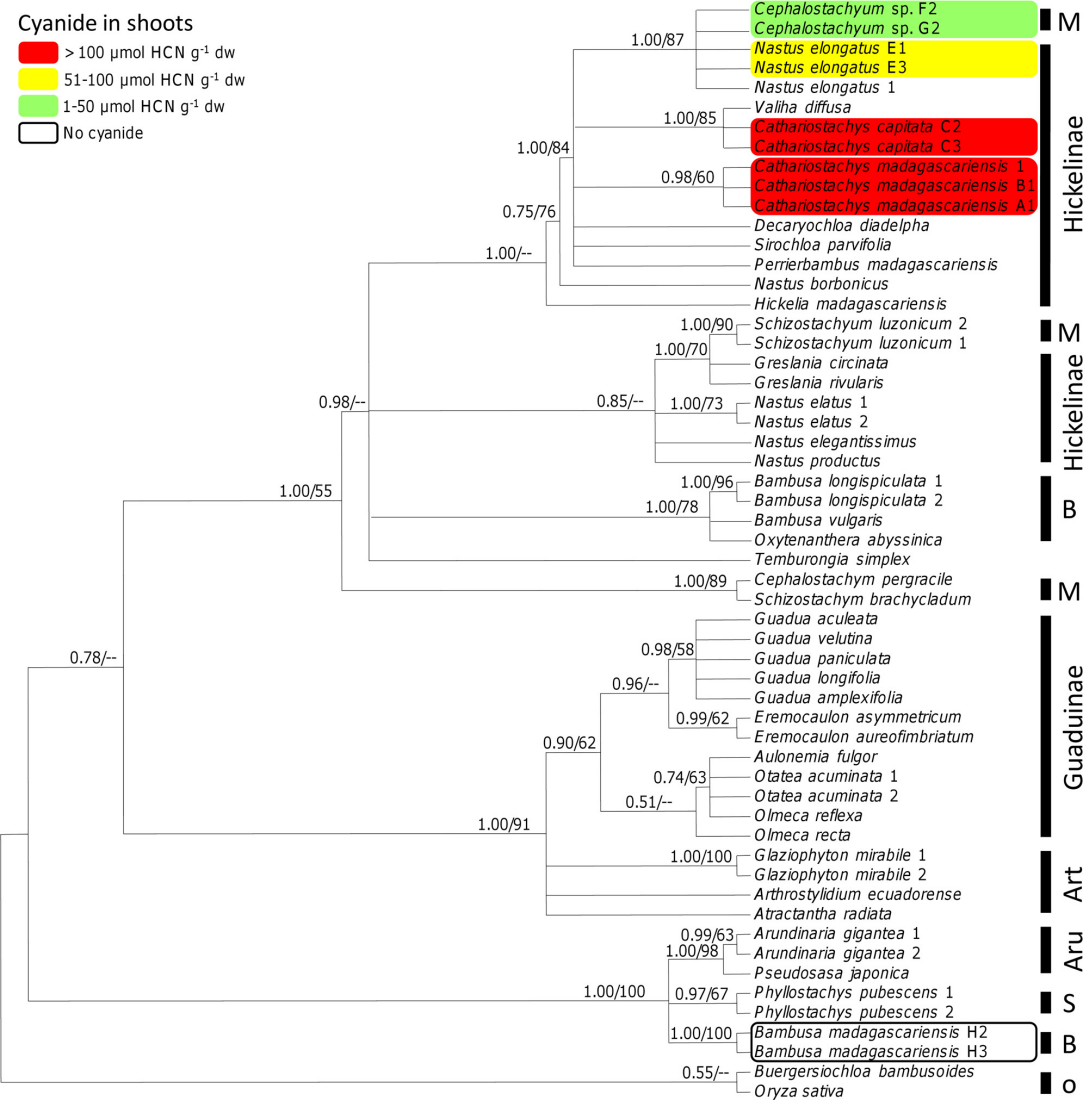
Molecular phylogeny of bamboo species based on rpl16 intron sequence data. This is a 50% majority rule consensus tree based on 150,000 trees from a B/MCMC tree sampling procedure. Posterior probabilities and ML bootstrap support values are indicated above branches (pp/ML BS). Cyanogenic potential (HCNp = concentration of cyanogenic precursors) of the woody bamboo species occurring in the Ranomafana area is drawn on the tree. Subtribes are indicated on the right of the figure: Art, Arthrostylidiinae; Aru, Arundinariinae; B, Bambusinae; M, Melocanninae; S, Shibataeinae; o, outgroup.

Since the topologies of the ML and B/MCMC analyses did not show any strongly supported conflicts, only the 50% majority-rule consensus tree of Bayesian tree sampling is shown. Those nodes that received strong support (i.e., posterior probability (pp) ≥ 0.95 in B/MCMC analysis as well as ML bootstrap ≥ 70%) in both the ML and Bayesian were considered significant (Fig 2). Based on our dataset, several genera (i.e., *Bambusa*, *Cephalostachyum*, *Nastus*) and subtribes (Bambusinae, Hickelinae, Melocanninae) are not supported as being monophyletic. Nevertheless, Malagasy Hickelinae and *Cephalostachyum* sp. (Melocanninae) were strongly supported as being monophyletic and appear to be derived within the woody bamboos (tribe Bambuseae). This clade contained all species that we tested positive for cyanide (i.e., *C*. *madagascariensis*, *C*. *capitata*, *Cephalostachyum* sp., *N*. *elongatus*). Within the clade of the Malagasy Hickelinae + *Cephalostachyum* sp., the taxa are not resolved and we cannot make any predictions of the evolutionary course within this group. However, the sympatric species *B*. *madagascariensis* (subtribe Bambusinae) tested negative for cyanide and appears at a basal position within the woody bamboos (Fig 2).

### Lemur Phylogeny and Ancestral States Reconstruction

**Fig 3:**
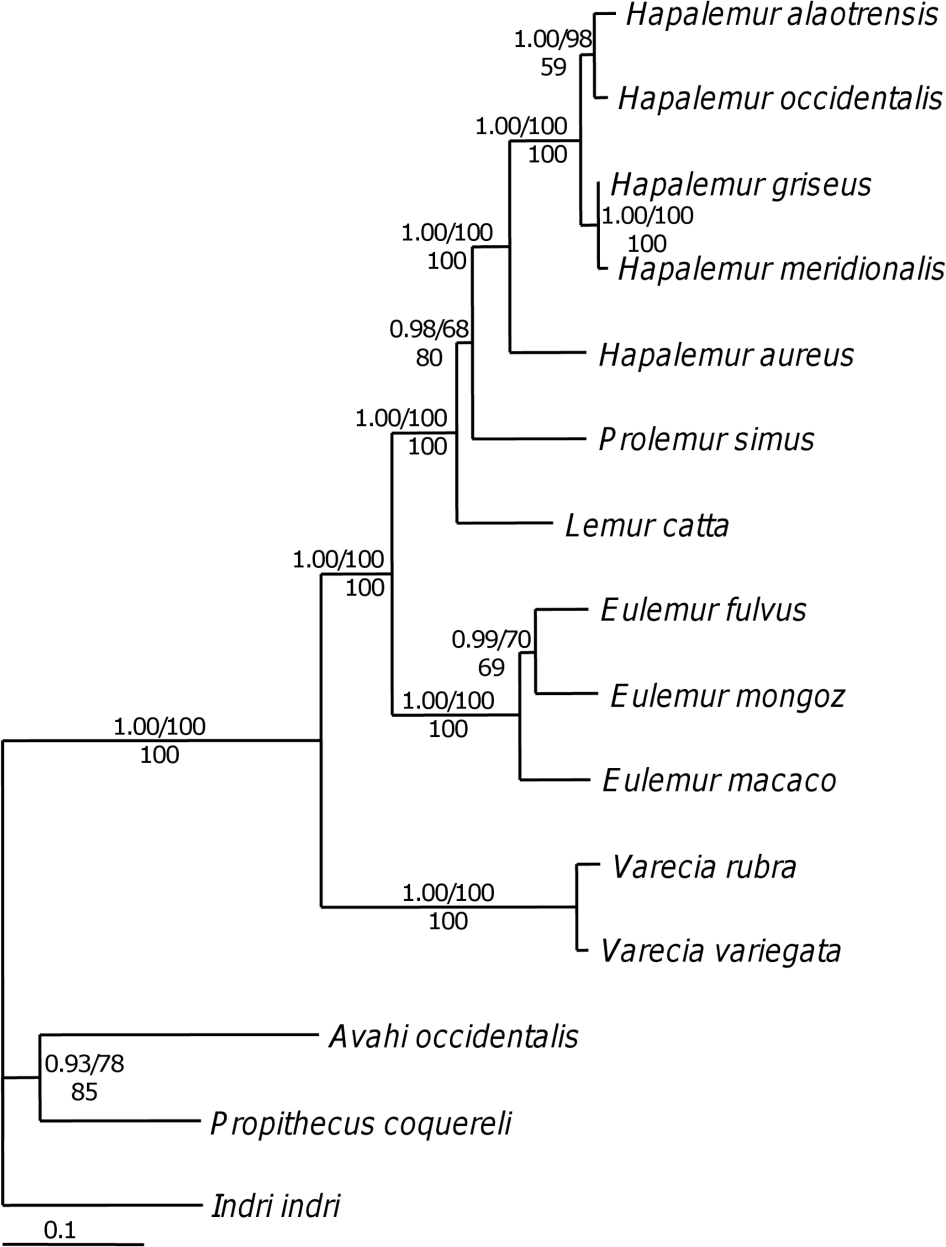
Phylogeny of bamboo lemurs as inferred from a five gene-partition analysis. The final alignment consisted of 5523 bp and included fragments of the 12s rRNA, cytochrome c oxidase subunit II (COII), cytochrome b (cyt-b), D-loop, as well as the Pastorini fragment (PAST) covering NADH3, NADH4L, NDH4 and 5 tRNAs. This is a 50% majority rule consensus tree based on 150,000 trees from a B/MCMC tree sampling procedure. Posterior probabilities and ML bootstrap support values are indicated above branches (pp/ML BS) while MP BS support values are given below branches.

To infer the molecular phylogeny of bamboo lemurs, we used 69 sequences of 15 lemur taxa (Table 2). The final alignment consisted of 5523 bp for the following five gene regions: A fragment of the 12S rDNA region (840 bp), the cytochrome c oxidase subunit II (COII) (684 bp), the cytochrome b (cyt b) (1140 bp), a portion of the D-loop fragment (465 bp), and the Pastorini fragment (PAST) (2394 bp). The combined alignment is available in TreeBASE (http://purl.org/phylo/treebase/phylows/study/TB2:S18996). Models for DNA substitution were estimated for each gene. The mean log likelihood of the Bayesian tree sampling was –25413.58. Detailed information on base composition and estimated parameter values for each partition is given in S3 Table. Maximum likelihood and maximum parsimony analyses of the combined data set yielded a tree that did not contradict the Bayesian tree topology and only the 50% majority-rule consensus tree of Bayesian tree sampling is shown (Fig 3). The maximum likelihood search in GARLI v0.94 [82] using the GTR+I+ Γ model, which was determined the best fit model for the combined dataset using Modeltest, resulted in one maximum likelihood tree with a lnL = -25633.2494. The bootstrap values recovered with the maximum likelihood criterion (ML bs) are included in Fig 3. Of all 5523 characters, 3406 were constant, 556 were parsimony-uninformative and 1561 parsimony-informative. The maximum parsimony (MP) analysis resulted in one most parsimonious tree with a length of 4533 steps, a CI of 0.5908 and a RI of 0.4029. The bootstrap values recovered with the maximum parsimony criterion (MP bs) are included in Fig 3.

In the majority-rule consensus tree of the combined data set shown in Fig 3, the family Lemuridae with the genera, *Eulemur*, *Hapalemur*, *Lemur*, *Prolemur* and *Varecia* is highly supported as being monophyletic. The sister relationship of *Eulemur* to *Lemur*, *Prolemur* and *Hapalemur* is strongly supported. The clade consisting of *Lemur*, *Prolemur*, and *Hapalemur* also has strong support. However, the monophyly of the bamboo lemur genera *Prolemur* and *Hapalemur* is only significantly supported in the Bayesian analysis (pp = 0.98) and MP (BS = 80) analysis, but and not in the ML analysis (BS = 68). The monophyly of *Hapalemur* is strongly supported in all three analyses.

**Fig 4:**
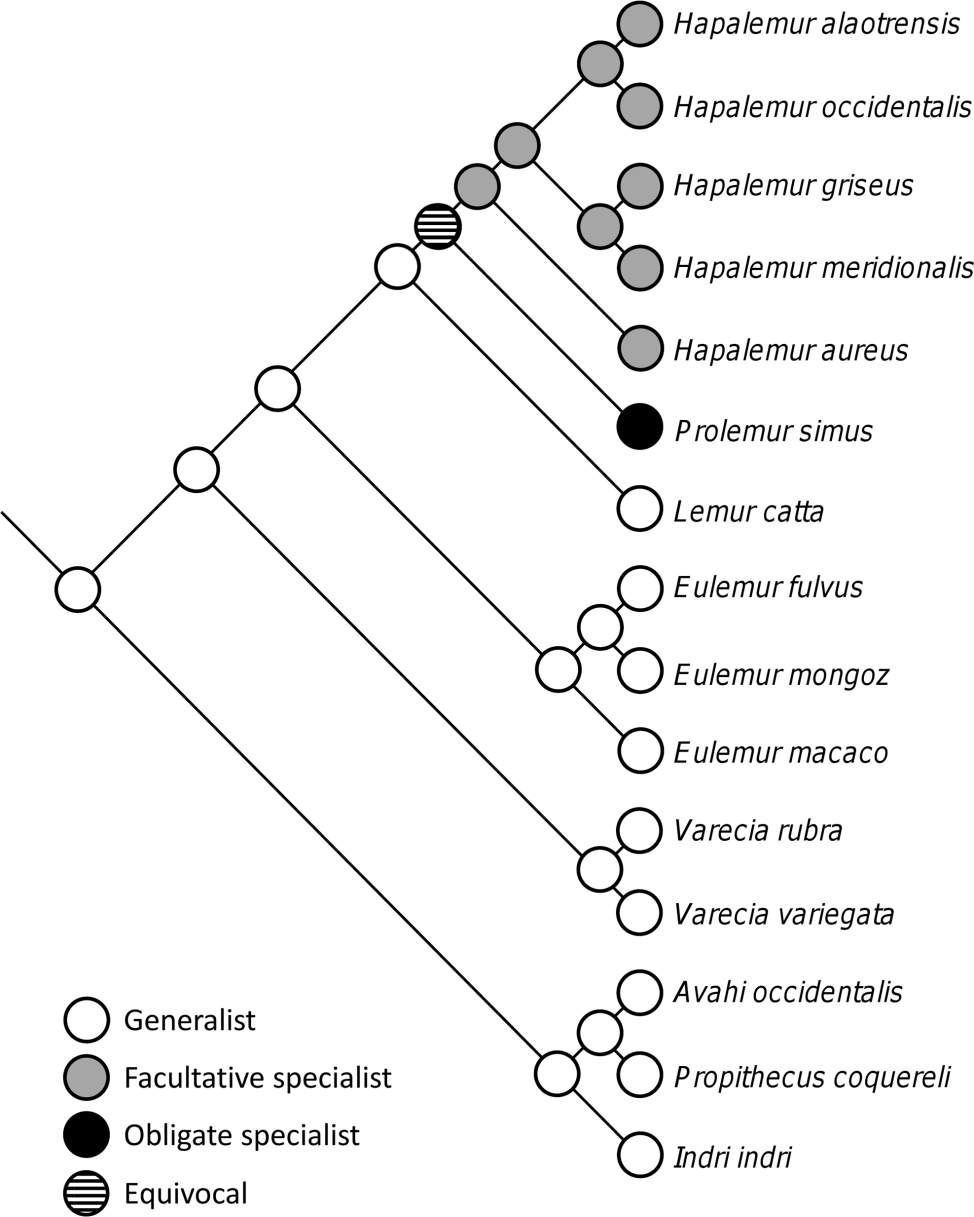
Lemur's degree of specialization towards bamboo plants traces on the phylogeny of lemurs, as inferred from a five gene fragments analysis. Possible states were "generalist" (either obligate of facultative), "facultative specialist" meaning that bamboo is the major food plant but other plants are also regularly consumed in nature and "obligate specialist" meaning that bamboo is the sole food plant accounting for more than 95% of the lemur's diet.

Among the 15 lemur species included in this study, nine were non-specialized (genera *Avahi*, *Eulemur*, *Indri*, *Lemur*, *Propithecus*, and *Varecia*), five showed an intermediate degree of food specialization (genus *Hapalemur*), and one was highly specialized (genus *Prolemur*). Ancestral character mapping of degree of specialization on the phylogeny (Fig 4) leads to the conclusion that the ancestors to lemurs were non-specialized, that specialization to bamboo food plants has arisen within the bamboo lemurs (genera *Hapalemur* and *Prolemur*) and was followed by evolution of more generalist life styles (*H*. *aloatrensis*, *H*. *occidentalis*, *H*. *griseus* and *H*. *meridionalis*). With the current data set, the state of the ancestor to the bamboo lemurs, however, remains unresolved. All three putative states cannot be rejected: i) the ancestor might have been non-specialized with high specialization arising once in *Prolemur* and low specialization once in *Hapalemur*. ii) The ancestor might have been a facultative specialist with obligate specialization arising in *Prolemur*. iii) The ancestor might have been an obligate specialist with facultative specialization arising in *Hapalemur*. Most importantly, however, we note that the facultative specialization occurs once in *Hapalemur* and that *Hapalemur* forms one monophyletic and species-rich group.

## Discussion

### Bamboo Cyanogenesis and Bamboo Lemur Coevolution

Plant-herbivore interactions can lead to escalating evolutionary arms-races in which plants express increasingly higher or more complex defenses in order to escape their antagonists, while herbivores develop more efficient counter-adaptations [15,94]. Our nutritional analyses of five Malagasy bamboo species sympatrically occurring in the island's southeast demonstrate that concentration of cyanogenic glycosides in shoots of giant bamboo (*C*. *madagascariensis*) reaches extreme levels compared to other cyanogenic plants [69] and is highest among the bamboo species investigated. Among the three co-occurring bamboo lemur species in the Ranomafana area, *P*. *simus* relies most heavily on cyanogenic food as it almost exclusively feeds on *C*. *madagascariensis*, the most cyanogenic bamboo [40,44]. However, exact data on the amount of cyanide that lemurs are actually exposed to are difficult to obtain as individual giant bamboo plants show quantitative variation in cyanide content [48] and feeding choice of lemurs might potentially be correlated to intraspecific variability of cyanogenesis in this plant [95]. Nevertheless, as giant bamboo overall represents an exceptionally cyanogenic plant, high levels of cyanide exposure can be assumed.

Although specialist herbivores are typically considered able to cope with toxins of their food plants, this does not necessarily mean that they are unaffected by quantitative variation of these compounds [48]. Detoxification processes often incur costs due to the expression of enzymes involved in degradation of toxins [10,96–98]. Thus, extremely high concentrations of toxins are likely to affect performance and reproduction—and ultimately fitness—even in specialist herbivores [19,99–101].

We found significant differences in cyanogenic potential among the bamboo species investigated ranging from extraordinary high levels in *C*. *madagascariensis* to no cyanide in *B*. *madagascariensis*. As we exclusively quantified cyanide in the bamboo species co-occurring in the Ranomafana area, we cannot draw general conclusions about the evolution of cyanogenesis in bamboos. Our results are largely consistent with previous phylogenies [93,102], but with the addition of several new taxa and specimens. Based on this dataset, the support of most branches is weak. Some genera (i.e., *Bambusa*, *Cephalostachyum*, *Nastus*) and subtribes (Bambusinae, Hickelinae, Melocanninae) are not supported as being monophyletic, which is consistent with earlier findings using the same sequence fragment [75]. It is, however, noteworthy that all Malagasy Hickelinae clustered together in the molecular phylogeny including the high cyanogenic species *C*. *madagascariensis*, *C*. *capitata*, *N*. *elongatus*, and the low cyanogenic *Cephalostachyum* sp. This indicates that cyanide concentration can vary substantially among closely related taxa [103,104]. At the same time, we found large variation within a single species (Fig 1). Our comparative quantitative data on cyanide concentrations in different sympatric bamboos collected from natural populations are the first of this kind. Once more taxa are analyzed for their cyanogenic potential and additional genes are sequenced across the entire bamboo phylogeny, we will be able to draw further conclusions on the evolution of cyanogenesis in woody bamboos.

In addition to toxic compounds, overall quality of food plants for herbivores is critically affected by the amount and composition of nutritive compounds [105,106]. In particular, the amount of protein essentially determines food quality [67,107,108]. Ganzhorn [67] reported an average protein concentration of 6.4% per dry weight in forest tree leaves in the Ranomafana area. Protein concentration in shoots of bamboo is considerably lower and ranges from 3.5–5.5% of dry weight [48] indicating that the bamboo diet of the lemurs might be nitrogen limited. The ratio of HCN per protein is of particular relevance for the nutritional quality of a given food plant as the major mechanism of cyanide detoxification in mammals—the conversion of cyanide to thiocyanate by activity of rhodanase—requires the presence of the S-containing amino acids methionine and cysteine [109,110]. Bamboo lemurs have been shown to excrete cyanide (likely in form of thiocyanate) in urine [111], however quantitative data are not available. Glander et al. [44] argued that based on nutritional data on Asian bamboo species, the concentrations of methionine and cysteine in *C*. *madagascariensis* are relatively low. However, the low concentration of S-containing amino acids, as observed in Asian bamboos which serve as browse for giant pandas [112], has not been analyzed for *C*. *madagascariensis* or other Malagasy bamboos.

### Specialization as an Evolutionary Dead End

It has been postulated that extreme specialization is an evolutionary dead-end leading to extinction [3,94,113–115]. In phylogenies, specialization should be of recent origin [116] as earlier origins of this trait should have ended in extinction [117]. It is also expected that generalists should be ancestral to specialists and not vice versa (generalists-to-specialist-hypothesis; [3,116,118]). Several studies support this hypothesis (reviewed in [119]). On the other hand, examples of ancestral specialization have been found in cowbirds [120] and in anthidiine bees [121].

In the current study, we found that the extremely specialized *Prolemur* is ancestral to the less specialized *Hapalemur*. It is also remarkable that once food plant specialization decreases in *Hapalemur*, rise is given to several species in different habitats across the entire island. *Hapalemur griseus* and its subspecies (which have recently been elevated to the species status *H*. *occidentalis*, *H*. *meridionalis*, *H*. *alaotrensis*, and *H*. *gilberti*; [47]) occur in various habitats ranging from littoral forests to swamps that contain little or no woody bamboos. Here the lemurs feed on a range of different plant species [42]. It seems that once the ties of being totally restricted to a single and extremely toxic host plant are removed, room is given for speciation and the exploitation of various habitats.

Thus, while our data strongly suggest that the ancestral state of lemurs most likely is a generalist, it is not clear whether from this generalist state a facultative or obligate specialist evolved first (as the common ancestor is equivocal) (Fig 4). Nevertheless, the fact that the non- to medium-specialized group is comprised of several lemur species (in contrast to *Prolemur* with only one extreme specialist) suggests an adaptive radiation, probably due to a more successful generalist life style.

### Advantages of Generalist Foraging Strategies

There are mainly two factors that favor generalist diets [11]: i) dietary mixing and ii) an increased availability of host plants. In the case of bamboo lemurs, we do not have the means to test performance of these primates in feeding experiments and we can only make assumptions. However, both factors seem plausible. Dietary mixing might be advantageous given the extreme levels of cyanide as found for *C*. *madagascariensis* and given the fact that high cyanide levels reduce herbivore performance, both for generalist and specialist herbivores [100,101,122]. Studies testing the performance of herbivores on single and mixed diets using insects found contradicting results. While for grasshoppers (Orthoptera), mixed diets usually lead to increased performance, no such effects were found for butterflies (Lepidoptera) and Hemiptera [11]. Positive effects of dietary mixing have also been found in several vertebrate taxa including mammals, birds, reptiles, and fish [5,10,123–132].

As outlined previously, we suggest that there are various reasons why specialization might be critical for herbivorous mammals. Indeed, extreme food plant specialization is rare in mammalian herbivores. Probably the most well-known examples of specialized herbivorous mammals are the giant (Ursidae: *Ailuropoda melanoleuca*) and red pandas (Ailuridae: *Ailurus fulgens*)—e.g., [133,134]—which feed on bamboos, as well as koalas (Phascolarctidae: *Phascolarctos cinereus*), which feed on a limited range of eucalypt food plants [135,136]. The bamboo lemurs from Madagascar represent another case of extreme specialization in herbivorous mammals. Interestingly, both pandas and lemurs are feeding on bamboos, and all three, pandas, lemurs, and koalas are feeding on cyanogenic food plants. However, there is little information available on the quantitative intake of cyanide in these species. With focus on bamboo lemurs, besides reduced intake of highly toxic cyanide when choosing alternative hosts, advantages of host plant availability for less specialized bamboo lemurs seem very likely. As mentioned previously, the colonization of habitats largely or completely lacking woody bamboo species as observed for example for *H*. *meridionalis* and *H*. *alaotrensis* is only possible as these species feed on alternative hosts [42].

### Consequences of Coevolutionary Processes

In mammalian herbivores, coevolutionary processes between food plant chemical defenses and herbivores leading to generalist or specialist foraging strategies have been little studied so far [9,137,138]. This is in sharp contrast to the detailed knowledge on the coevolution of plant defenses and other herbivore groups such as herbivorous insects. In mammalian and insect herbivores, variation in rates of coevolution should be expected. Insects, for example, often show large numbers of offspring and short reproduction cycles and thus inherently exhibit faster evolution. This situation however should be different in mammals mostly showing slower rates of reproduction and producing less offspring than insects. Due to lower rates of evolution, highly specialized mammals should experience higher risks of reaching evolutionary dead ends and extinction, whereas in specialist herbivorous insects host shifts are common and have been frequently reported—e.g., [139–141].

From the mammals’ perspective, extreme specialization includes several high risks. Specialist mammalian herbivores relying on a single food cannot rapidly evolve new traits (as compared to insect herbivores with their short generation cycles), which makes host shifts difficult. Furthermore, herbivorous mammals specializing on highly toxic plants such as the bamboo lemurs may be trapped in an evolutionary dead end as food plants develop levels of toxic compounds that reach physiologically tolerable thresholds in the herbivore. This can either be due to evolving higher concentrations of these compounds but also can occur due to the phenotypic plasticity of plants in response to environmental factors. In our previous studies, we demonstrated phenotypically increased levels of cyanide in lima bean (*Phaseolus lunatus*) in response to drought stress and increasing soil salinity [49]. In feeding experiments with specialist insect herbivores (Mexican bean beetle; *Epilachna varivestis*), enhanced cyanogenic features significantly decreased the amount of plant material consumed and the reproduction of herbivores over multiple generations indicating quantitative costs of cyanogenesis even for specialist herbivores [101,102]. The fact that insects, which on population levels should respond much faster to changes in food plant quality than herbivorous mammals, show distinct impairment suggests that even more substantial effects of increased cyanogenesis should be observed on slowly reproducing mammalian herbivores.

## Conclusions

The coevolutionary adaptation of herbivores and increasing chemical defense of plants can potentially lead to an ultimate threshold of toxin that does not allow for further physiological adaptation of herbivores. As long as no host switches occur, specialists would be trapped in an insolvable situation as they rely on a single food source. In contrast, the evolution of a more generalist foraging strategy allows for escape from the escalating evolutionary arms race of enhanced defense and increased counter-adaptation as well as the colonization of new and less limited habitats. The plant-herbivore system consisting of different bamboo species with different degrees of toxicity and lemurs with different degrees of specialization to this toxicity represents a unique opportunity to understand the sources and outcomes of coevolutionary processes in mammals.

## Supporting Information

S1 Table Voucher and collection data of bamboo plants included in the phylogenetic study. Material was collected by Daniel. J. Ballhorn, Stefanie Kautz, Fanny P. Rakotoarivelo, Georges Razafindrakoto. Vouchers are deposited at Portland State University.
(DOCX)

S2 Table Parameters of Bayesian tree sampling (Bamboo phylogeny). (DOCX)

S3 Table Parameters of Bayesian tree sampling of data partitions (lemur phylogeny). (DOCX)
