# Supplementary material for: Coevolution of Cyanogenic Bamboos and Bamboo Lemurs on Madagascar
Source: PLoS One. 2016 Aug 17;11(8):e0158935. doi: 10.1371/journal.pone.0158935 (PMC4988758; doi:10.1371/journal.pone.0158935)
Supplement: S1 Table — Material was collected by Daniel. J. Ballhorn, Stefanie Kautz, Fanny P. Rakotoarivelo, Georges Razafindrakoto. Vouchers are deposited at Portland State University. (DOCX) [file pone.0158935.s001.docx]

S1 Table. Voucher and collection data of bamboo plants included in the phylogenetic study. Material was collected by Daniel. J. Ballhorn, Stefanie Kautz, Fanny P. Rakotoarivelo, Georges Razafindrakoto. Vouchers are deposited at Portland State University.

| Specimen | Voucher | GPS data | Date collected |
| --- | --- | --- | --- |
| *Bambusa madagascariensis* H2 | DB | 21°14.447 S  047°23.544 E  elevation 1134 m | Feb-02-2008 |
| *Bambusa madagascariensis* H3 | DB | 21°14.851 S  047°22.804 E  elevation 1151 | Feb-02-2008 |
| *Cathariostachys madagascariensis* A1 | DB | 21°14.991 S  047°25.176 E  elevataion 1121 m | Jan-21-2008 |
| *Cathariostachys madagascariensis* B1 | DB | 21°14.923 S  047°25.148 E  elevation 1110 m | Jan-23-2008 |
| *Cathariostachys capitata* C2 | DB | 21°21.381 S  047°47.304 E  elevation 206 m | Jan-25-2008 |
| *Cathariostachys capitata* C3 | DB | 21°21.409 S  047°46.832 E  elevation 222 m | Jan-25-2008 |
| *Cephalostachyum sp.* F2 | DB | 21°14.404 S  047°23.525 E  elevation 1137 m | Feb-02-2008 |
| *Cephalostachyum sp.* G2 | DB | 21°15.054 S  047°25.561 E  elevation 890 m | Feb-02-2008 |
| *Nastus elongatus* E1 | DB | 21°15.181 S  047°24.162 E  elevation 1058 m | Jan-28-2008 |
| *Nastus elongatus* E2 | DB | 21°15.235 S  047°24.216 E  elevation 1037 m | Feb-02-2008 |
