# Supplementary material for: Coevolution of Cyanogenic Bamboos and Bamboo Lemurs on Madagascar
Source: PLoS One. 2016 Aug 17;11(8):e0158935. doi: 10.1371/journal.pone.0158935 (PMC4988758; doi:10.1371/journal.pone.0158935)
Supplement: S2 Table — (DOCX) [file pone.0158935.s002.docx]

|  |  |
| --- | --- |
| Statistic | Mean |
| πA_mean/all_ | 0.393 |
| πC_mean/all_ | 0.142 |
| πG_mean/all_ | 0.177 |
| πT_mean/all_ | 0.288 |
| r(AC) _mean/all_ | 0.150 |
| r(AG) _mean/all_ | 0.199 |
| r(AT) _mean/all_ | 0.079 |
| r(CG) _mean/all_ | 0.095 |
| r(CT) _mean/all_ | 0.366 |
| r(GT) _mean/all_ | 0.111 |
| α_mean/all_ | 0.097 |

S2 Table. Parameters of Bayesian tree sampling (Bamboo phylogeny).
