# Supplementary material for: Coevolution of Cyanogenic Bamboos and Bamboo Lemurs on Madagascar
Source: PLoS One. 2016 Aug 17;11(8):e0158935. doi: 10.1371/journal.pone.0158935 (PMC4988758; doi:10.1371/journal.pone.0158935)
Supplement: S3 Table — (DOCX) [file pone.0158935.s003.docx]

S3 Table. Parameters of Bayesian tree sampling of data partitions (lemur phylogeny).

|  | Partition 1 | Partition 2 | Partition 3 | Partition 4 | Partition 5 | Partition 6 | Partition 7 | Partition 8 | Partition 9 |
| --- | --- | --- | --- | --- | --- | --- | --- | --- | --- |
| Gene partition | 12S | mtCOII  1^st^ position | mtCOII  2^nd^ position | mtCOII 3^rd^ position | cyt-b  1^st^  position | cyt-b  2^nd^ position | cyt-b  3^rd^ position | D-loop | PAST |
| πA_mean/all_ | 0.366 | 0.268 | 0.255 | 0.400 | 0.315 | 0.202 | 0.376 | 0.310 | 0.339 |
| πC_mean/all_ | 0.232 | 0.256 | 0.251 | 0.253 | 0.276 | 0.253 | 0.377 | 0.133 | 0.286 |
| πG_mean/all_ | 0.175 | 0.249 | 0.114 | 0.059 | 0.194 | 0.133 | 0.034 | 0.264 | 0.010 |
| πT_mean/all_ | 0.226 | 0.227 | 0.380 | 0.288 | 0.214 | 0.411 | 0.214 | 0.293 | 0.276 |
| r(AC) _mean/all_ | 0.030 | 0.041 | 0.036 | 0.020 | 0.019 | 0.066 | 0.074 | N/A | 0.029 |
| r(AG) _mean/all_ | 0.146 | 0.213 | 0.248 | 0.519 | 0.331 | 0.483 | 0.573 | N/A | 0.346 |
| r(AT) _mean/all_ | 0.083 | 0.042 | 0.051 | 0.015 | 0.022 | 0.028 | 0.013 | N/A | 0.036 |
| r(CG) _mean/all_ | 0.013 | 0.082 | 0.183 | 0.013 | 0.018 | 0.040 | 0.062 | N/A | 0.016 |
| r(CT) _mean/all_ | 0.705 | 0.675 | 0.435 | 0.420 | 0.574 | 0.342 | 0.276 | N/A | 0.568 |
| r(GT) _mean/all_ | 0.023 | 0.021 | 0.048 | 0.012 | 0.043 | 0.040 | 0.070 | N/A | 0.010 |
| α _mean/all_ | 0.416 | 0.413 | 0.073 | 2.419 | 0.617 | 0.134 | 3.119 | 0.847 | 1.105 |
| P(invar) _mean/all_ | 0.399 | 0.695 | 0.569 | 0.022 | 0.561 | 0.762 | 0.018 | 0.376 | 0.495 |
